# Supplementary figures and images for: Effects of Fine Particulate Matter on Cardiovascular Disease Morbidity: A Study on Seven Metropolitan Cities in South Korea
Source: Int J Public Health. 2022 May 16;67:1604389. doi: 10.3389/ijph.2022.1604389 (PMC9149776; doi:10.3389/ijph.2022.1604389)

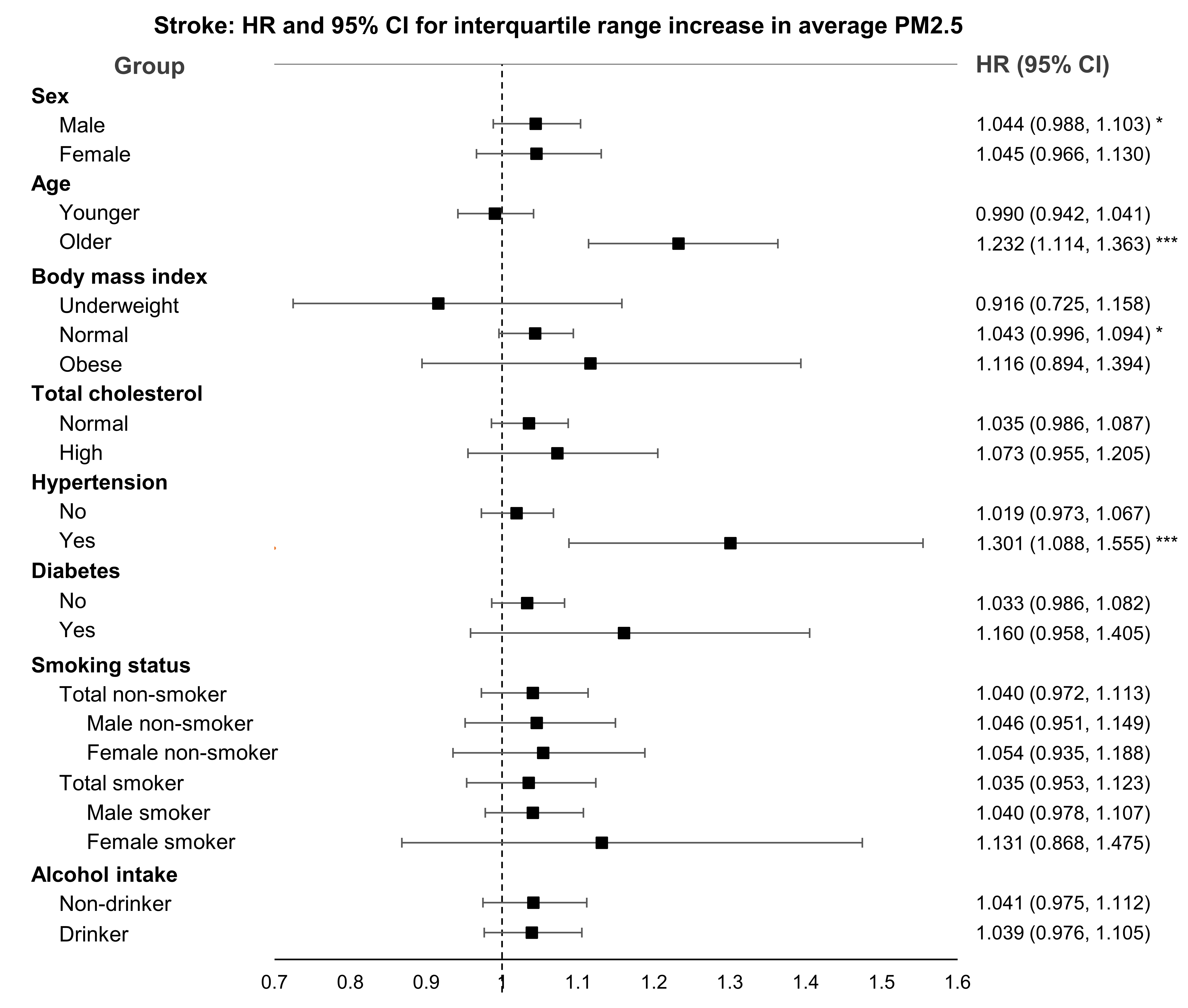

Supplement: Supplementary file 1 [file Image3.TIF]

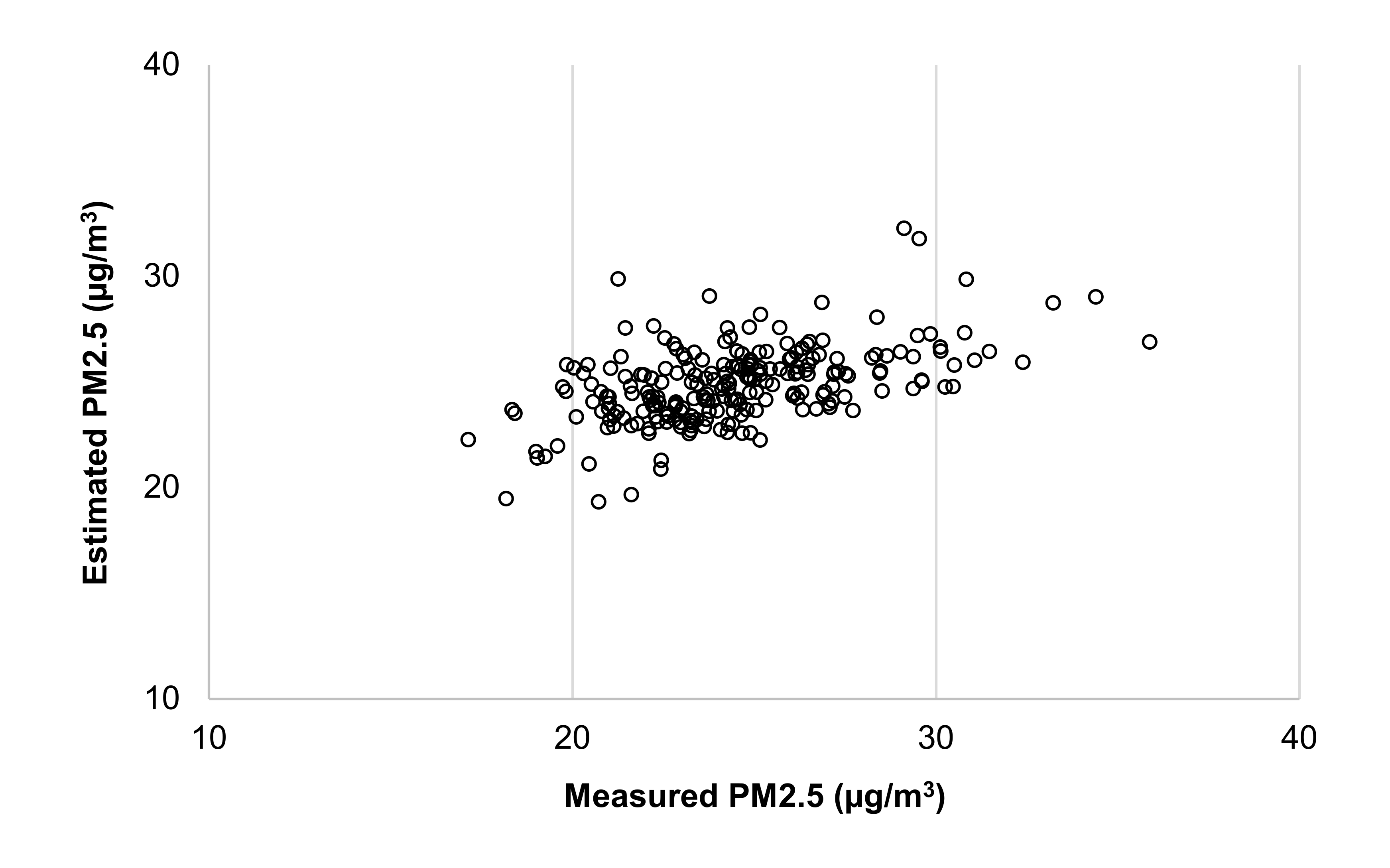

Supplement: Supplementary file 2 [file Image2.TIF]

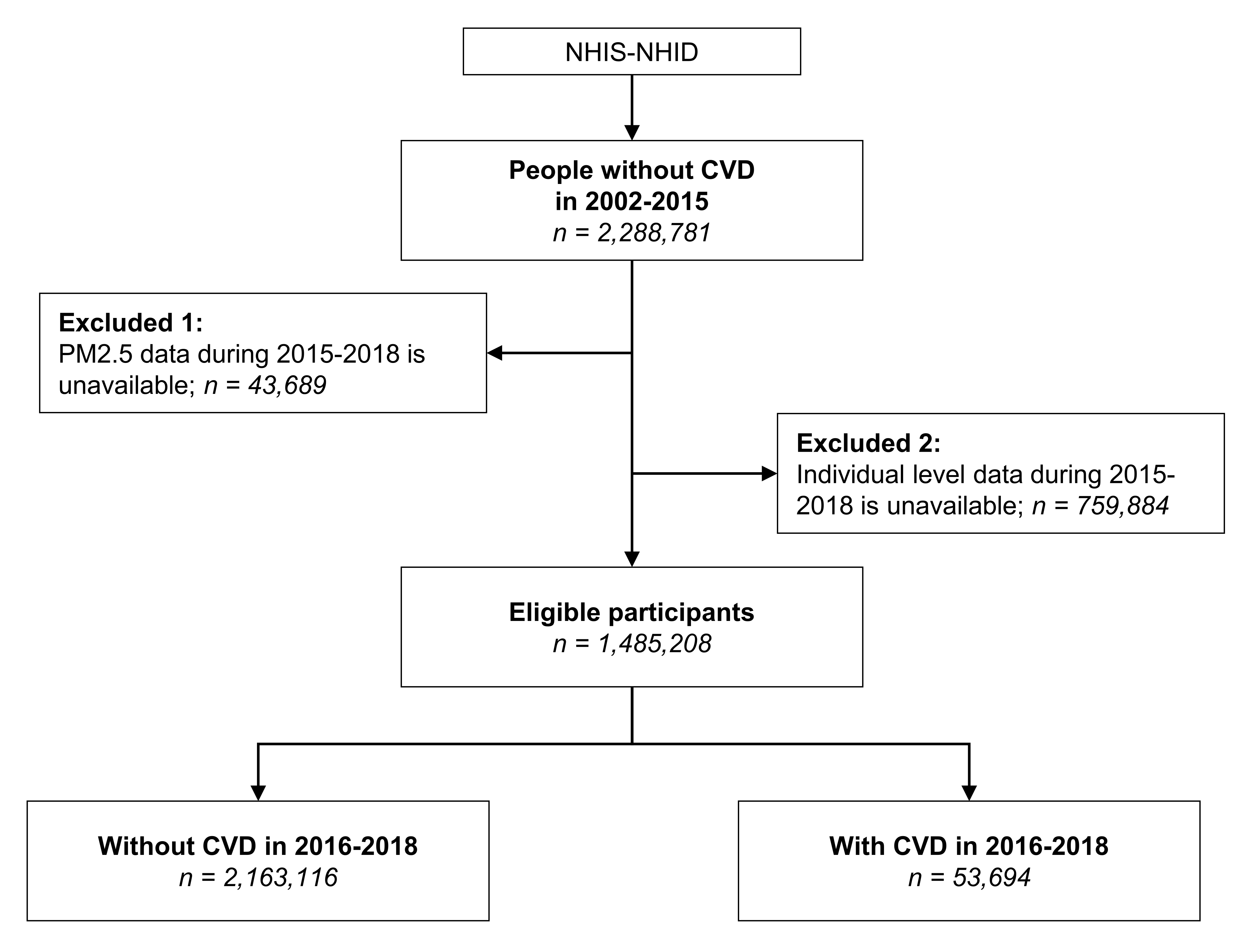

Supplement: Supplementary file 3 [file Image1.TIF]
