# Supplementary material for: Effects of Fine Particulate Matter on Cardiovascular Disease Morbidity: A Study on Seven Metropolitan Cities in South Korea
Source: Int J Public Health. 2022 May 16;67:1604389. doi: 10.3389/ijph.2022.1604389 (PMC9149776; doi:10.3389/ijph.2022.1604389)
Supplement: Supplementary file 4 [file DataSheet1.docx]

Table S1. Particulate matter alerts trends by metropolitan city in South Korea between 2016 and 2019 (days)

| **Region** | **2016** | **2017** | **2018** | **2019** |
| --- | --- | --- | --- | --- |
| Seoul | 0 | 10 | 18 | 29 |
| Busan | 10 | 6 | 11 | 16 |
| Daegu | 0 | 4 | 6 | 16 |
| Incheon | 3 | 17 | 22 | 28 |
| Gwangju | 2 | 12 | 15 | 20 |
| Daejeon | 1 | 2 | 10 | 17 |
| Ulsan | 0 | 2 | 6 | 11 |

Table S2. Characteristics of all and selected participants [National Health Insurance Service–National Health Information Database, South Korea, 2002–2018]

| **Characteristics** | **All participants** | **Selected participants** |
| --- | --- | --- |
| **Total [n (%)]** | 2,245,092 (100) | 1,485,208 (100) |
| **Sex [n (%)]** | | |
| Male | 1,226,710 (54.6) | 856,017 (57.6) |
| Female | 1,018,382 (45.4) | 629,191 (42.4) |
| **Age [n (%)]** | | |
| Under 30 years | 354,043 (15.8) | 171,971 (11.6) |
| 30–40 years | 576,888 (25.7) | 395,969 (26.7) |
| 40–50 years | 652,665 (29.1) | 470,224 (31.7) |
| 50–60 years | 445,495 (19.8) | 304,871 (20.5) |
| 60–70 years | 170,293 (7.6) | 116,922 (7.9) |
| Above 70 years | 45,708 (2) | 25,251 (1.7) |
| Average age ^a^ | 43.8 (12.17) | 44.6 (11.42) |

^a^ Data is presented as mean (standard deviation)

Table S3. The number of particulate matter monitoring stations by city and province in South Korea between 2015 and 2018

| **Region** | **2015** | **2016** | **2017** | **2018** |
| --- | --- | --- | --- | --- |
| Total | 125 | 181 | 242 | 328 |
| Seoul | 25 | 25 | 25 | 25 |
| Busan | 19 | 19 | 19 | 20 |
| Daegu | 7 | 7 | 11 | 13 |
| Incheon | 8 | 14 | 15 | 17 |
| Gwangju | 6 | 6 | 7 | 7 |
| Daejeon | 1 | 6 | 8 | 10 |
| Ulsan | 6 | 6 | 7 | 16 |
| Sejong | 0 | 2 | 2 | 4 |
| Gyeonggi | 15 | 31 | 60 | 81 |
| Gangwon | 4 | 6 | 7 | 11 |
| Chungbuk | 9 | 10 | 11 | 13 |
| Chungnam | 1 | 5 | 19 | 27 |
| Jeonbuk | 3 | 12 | 12 | 23 |
| Jeonnam | 6 | 11 | 13 | 22 |
| Gyeongbuk | 1 | 7 | 10 | 13 |
| Gyeongnam | 11 | 11 | 12 | 21 |
| Jeju | 3 | 3 | 4 | 5 |

**Supplementary Figure Captions**

Figure S1 Data selection process for analysis [National Health Insurance Service–National Health Information Database, South Korea, 2002–2018]

Abbreviation: PM2.5, particulate matter less than 2.5μm in diameter

Figure S2 Plots of measured values at monitoring stations and estimated values by the Ordinary Kriging method in South Korea between 2015 and 2017

Abbreviation: PM2.5, particulate matter less than 2.5μm in diameter

Figure S3 Hazard ratios and 95% confidence intervals for stroke by subgroups [National Health Insurance Service–National Health Information Database, South Korea, 2002–2018]

Abbreviation: HR, Hazard Ratios; CI, Confidence Interval; PM2.5, particulate matter less than 2.5μm in diameter

Interquartile range was 2.9 µg/m^3^.

^*^ Significant at 10% level. ^***^ Significant at 1% level.
